# A Pathophysiological Approach to Spontaneous Orbital Meningoceles: Case Report and Systematic Review

**DOI:** 10.3390/jpm14050465

**Published:** 2024-04-28

**Authors:** Piergiorgio Gaudioso, Elia Biancoli, Veronica Battistuzzi, Stefano Concheri, Tommaso Saccardo, Sebastiano Franchella, Giacomo Contro, Stefano Taboni, Elisabetta Zanoletti, Francesco Causin, Lorena Nico, Joseph Domenico Gabrieli, Roberto Maroldi, Piero Nicolai, Marco Ferrari

**Affiliations:** 1 Section of Otorhinolaryngology-Head and Neck Surgery, Department of Neurosciences, “Azienda Ospedale Università di Padova”, University of Padua, 35128 Padua, Italy; piergiorgio.gaudioso@phd.unipd.it (P.G.); elia.biancoli@studenti.unipd.it (E.B.); veronica.battistuzzi@aopd.veneto.it (V.B.); stefano.concheri@studenti.unipd.it (S.C.); tommaso.saccardo@unipd.it (T.S.); sebastiano.franchella@aopd.veneto.it (S.F.); giacomo.contro@aopd.veneto.it (G.C.); stefano.taboni@aopd.veneto.it (S.T.); elisabetta.zanoletti@unipd.it (E.Z.); piero.nicolai@unipd.it (P.N.); 2 Unit of Otorhinolaryngology—Head and Neck Surgery, Azienda Ospedale Università Padova, 35128 Padua, Italy; 3 Oncology and Immunology (PhD Program), Department of Surgery Oncology and Gastroenterology (DiSCOG), University of Padova, 35128 Padova, Italy; 4 Technology for Health (PhD Program), Department of Information Engineering, University of Brescia, 25123 Brescia, Italy; 5 Artificial Intelligence in Medicine and Innovation in Clinical Research and Methodology (PhD Program), Department of Clinical and Experimental Sciences, University of Brescia, 25123 Brescia, Italy; 6 Section of Neuroradiology, Department of Diagnostic Imaging and Interventional Radiology, “Azienda Ospedale Università di Padova”, University of Padua, 35128 Padua, Italy; francesco.causin@aopd.veneto.it (F.C.); lorena.nico@aopd.veneto.it (L.N.); josephdomenico.gabrieli@aopd.veneto.it (J.D.G.); 7 Division of Radiology, Department of Medical and Surgical Specialties, Radiological Sciences and Public Health, University of Brescia, 25123 Brescia, Italy; roberto.maroldi@unibs.it; 8 Guided Therapeutics (GTx) Program International Scholarship, University Health Network (UHN), Toronto, ON M5G 2C4, Canada

**Keywords:** meningocele, meningoencephalocele, skull base, orbit, intracranial hypertension

## Abstract

Background: Spontaneous orbital cephaloceles are a rare condition. The purpose of this study is to provide a description of a clinical case and to carry out a systematic literature review. Methods: A systematic review of the English literature published on the Pubmed, Scopus, and Web of Science databases was conducted, according to the PRISMA recommendations. Results: A 6-year-old patient was admitted for right otomastoiditis and thrombosis of the sigmoid and transverse sinuses, as well as the proximal portion of the internal jugular vein. Radiological examinations revealed a left orbital mass (22 × 14 mm) compatible with asymptomatic orbital meningocele (MC) herniated from the superior orbital fissure (SOF). The child underwent a right mastoidectomy. After the development of symptoms and signs of intracranial hypertension (ICH), endovascular thrombectomy and transverse sinus stenting were performed, with improvement of the clinical conditions and reduction of the orbital MC. The systematic literature review encompassed 29 publications on 43 patients with spontaneous orbital MC. In the majority of cases, surgery was the preferred treatment. Conclusions: The present case report and systematic review highlight the importance of ICH investigation and a pathophysiological-oriented treatment approach. The experiences described in the literature are limited, making the collection of additional data paramount.

## 1. Introduction

Cephaloceles represent extracranial herniations of neural tissues through a defect in the skull. They can be classified as meningoceles (MC) when the herniation involves only the meningeal membranes, or as meningoencephaloceles (MEC) when brain parenchyma is involved [1]. Depending on the location of the defect, MC/MEC have been classified by Suwanwela et al. into the occipital, cranial vault, frontoethmoidal, basal, and cranioschisis types [2]. They can occur spontaneously or be the consequence of a trauma. Spontaneous cephaloceles can be congenital (presenting either shortly after birth or, in some instances, manifesting in adulthood [3]) or acquired when a medical condition promotes herniation through weakened areas of the skull base or foramina.

Orbital cephaloceles are a rare subtype of basal MC/MEC [2]. Patients may experience symptoms such as proptosis, visual impairment [4], and pulsatile proptosis [5]. Diagnosis mainly relies on radiology, with gadolinium-enhanced magnetic resonance (MR) being the gold-standard examination, allowing differential diagnosis with orbital cystic lesions, intra-orbital tumors, and vascular malformations [6,7,8]. High-resolution bone computed tomography (CT) can be useful in the identification of the herniation point, especially when a bony defect is implied. Considering the rarity of this condition, the optimal treatment strategy for orbital MC/MEC is tailored on a case-by-case basis, which can include watch-and-scan surveillance, medical therapy, and/or surgery [9,10,11,12,13,14].

In this study, a case report of spontaneous orbital MC is described. A systematic review of the literature is also performed to analyze the existing body of evidence and to investigate the role of intracranial hypertension (ICH) in the development and management of orbital cephaloceles.

## 2. Materials and Methods

### 2.1. Case Report

The patient’s clinical documentation was reviewed to formulate the case report.

### 2.2. Systematic Review

A systematic review of the English literature published on the Pubmed, Scopus, and Web of Science databases was conducted, according to the Preferred Reporting Items for Systematic Reviews and Meta-Analyses (PRISMA) recommendations [15]. The literature search was performed querying the following keywords: “orbital”, “orbit”, “cephalocele”, meningoencephalocele”, and “meningocele”. Keywords were combined when searching the aforementioned databases. Reference lists of all publications were also screened. The last search was performed on 17 January 2024. Our study is being registered in PROSPERO.

Investigations were included only if all the following criteria were met: (i) studies published in English in peer-reviewed journals, (ii) studies which describe spontaneous cephalocele (MC or MEC), (iii) studies with the respected characteristics of ethical compliance and completeness in presentation in accordance with the Case Reports (CARE) guidelines [16,17] and the Joanna Briggs Institute (JBI) checklist (Supplementary Table S1) [18]. Exclusion criteria were: (i) inaccessibility to full text, (ii) articles in the form of editorials, surveys, or letters to the editor, (iii) non-human model or cadaveric studies, (iv) studies not pertinent to spontaneous orbital MC or MEC, and (v) studies lacking relevant clinical data (clinical presentation, diagnostic assessment, and treatment). Any possible disagreements about the inclusion/exclusion of investigations and their quality were resolved by a discussion among the study team members.

Included studies were analyzed to extract available data and ensure eligibility for all patients. Data extracted from each study included the symptoms, ICH assessment, herniation point of the cephalocele, surgical treatment performed (if any), medical therapy, the outcome of ocular function, and follow-up. The risk of bias was assessed for all included studies.

## 3. Results

### 3.1. Case Report

**Figure 1:**
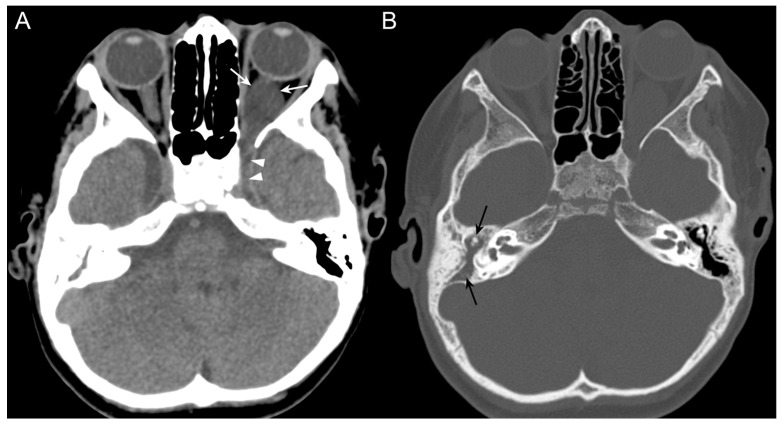
(**A**): CT without contrast agent administration shows an intra-orbital retrobulbar fluid-density sac (white arrows) on the left side. Posteriorly the fluid-filled sac replaces the fat tissue in the superior orbital fissure. The left cavernous sinus (arrowheads) is filled by a low density, similar to the intra-orbital sac. (**B**): the right middle ear is filled with fluid (black arrows).

A 6-year-old child (body weight 26 kg, height 120 cm, no significant medical history) presented to the pediatric emergency department of our Institution for episodes of vomiting (12 episodes in a day) and right otalgia. In the previous days, no fever, ocular symptoms, or headache were referred. The otoscopic assessment revealed signs of purulent right otitis media, with no eardrum perforation. Laboratory blood examination showed mild neutrophilic leukocytosis (12.31 × 10^9^/L leukocytes), and elevated C-reactive protein (152.60 mg/L). Intravenous antibiotic therapy (ceftriaxone, 100 mg/kg/day divided into two daily doses) was administered during the first day, and the child was kept under observation. The following day, the patient developed mild left eye proptosis, eyelid ptosis with periorbital edema, and drowsiness. At ophthalmologic examination, bilateral papilledema was observed (more pronounced on the left side). CT was performed (Figure 1), revealing an opacification of the right middle ear and mastoid cells and, collaterally, a 22 × 14 mm hypodense lesion in the retrobulbar intraconal space of the left orbit involving the orbital apex. 

**Figure 2:**
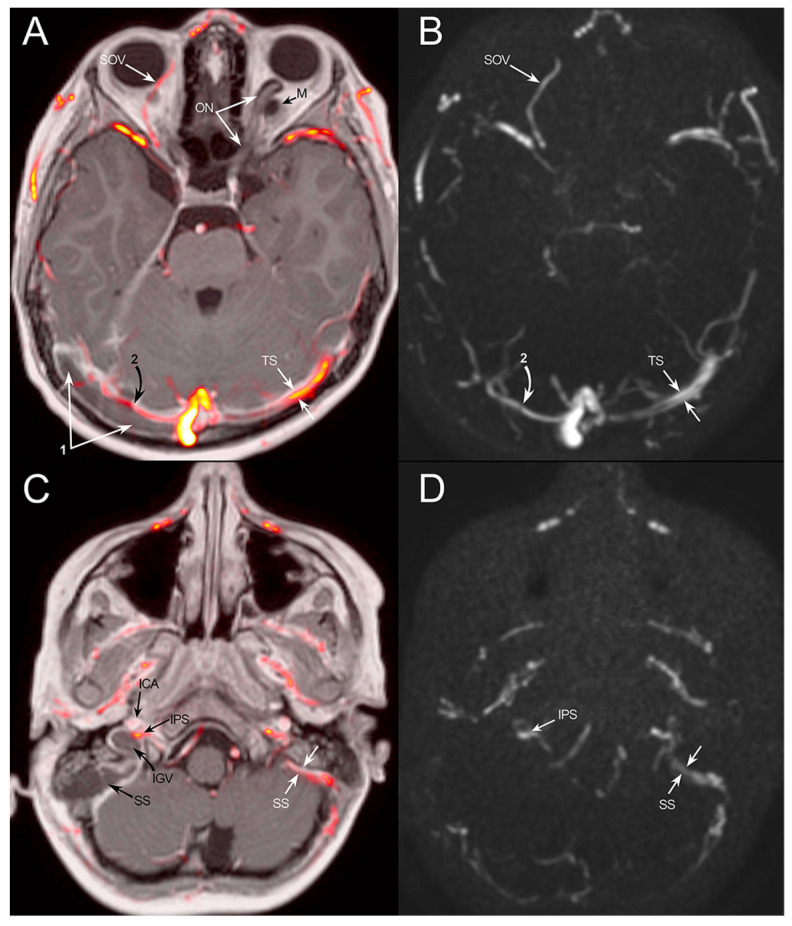
Post-contrast MR fused with MR venography (**A**–**C**) and MR venography (**B**–**D**). (**A**,**B**): thrombosis of the right transverse sinus (1), venography detects residual flow along its anterior wall (2). The left transverse sinus (TS) is hypoplastic. A prominent superior ophthalmic vein is shown on the right side (SOV). The left optic nerve (ON) is displaced by the intraorbital meningocele (M). (**C**,**D**): Fluid collection at the right mastoid with thrombosis of the sigmoid sinus (SS) and internal jugular vein (IJV). The right inferior petrosal sinus is patent (IPS). ICA: internal carotid artery.

This finding was further investigated with a contrast-enhanced angiographic MR (Figure 2), which revealed signs of otomastoiditis and right venous thrombosis involving the transverse sinus, sigmoid sinus, and proximal internal jugular vein. 

**Figure 3:**
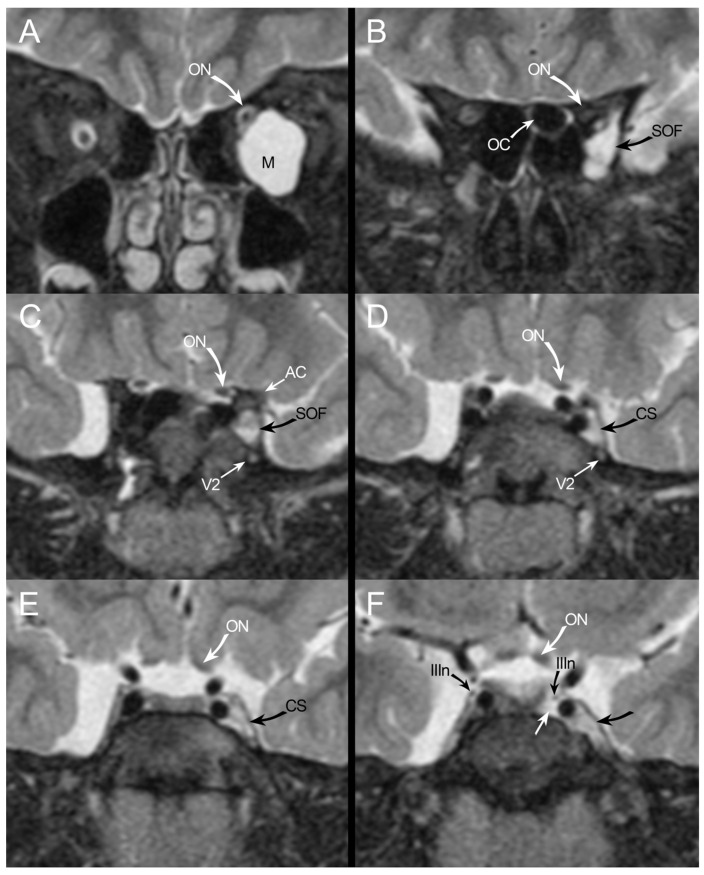
(**A**–**F**): The coronal T2 sequence shows the intraorbital meningocele (M) displacing the optic nerve (ON) upward and medially (**A**). A prominent subarachnoid space at the level of both optic sheaths is present. The meningocele enters the orbit via the superior orbital fissure (SOF) (**B**). At the anterior aspect of the cavernous sinus (**C**), the meningocele runs below the anterior clinoid (AC) and above the maxillary groove of the maxillary nerve (V2); it displaces the lateral wall of the cavernous sinus (**D**–**F**), causing a more convex shape (CS). At the posterior aspect of the cavernous sinus, the fluid signal is present both medially to the left internal carotid artery (white arrow) and laterally. The left oculomotor nerve runs in a lower position (IIIn) than the right one.

The left transverse sinus was found to be hypoplastic. Additionally, a 26 × 20 mm intraconal lesion was found in the left orbit (Figure 3), with homogeneous fluid content and without contrast enhancement. 

The lesion showed a rat’s tail in the orbital apex towards the parasellar area, displacing the optic nerve (ON) superiorly and impressing both the inferior and medial rectus muscles. The Fluid Attenuated Inversion Recovery (FLAIR) sequence showed a hypointense signal of the orbital lesion that was coherent with the hypothesis of MC herniated through the superior orbital fissure (SOF). In light of radiological findings, coagulation disorders were investigated, revealing a heterozygous mutation in the gene for Factor V Leiden.

Medical therapy with unfractionated heparin (100 IU/kg bolus, then 10–15 IU/kg/h), acetazolamide (38.5 mg/kg/day), vancomycin (60 mg/kg/day), and metronidazole (30 mg/kg/day) was administered. Subsequently, the patient underwent right mastoidectomy (with no evidence of purulent mastoiditis) and placement of a ventilation tube in the right ear.

**Figure 4:**
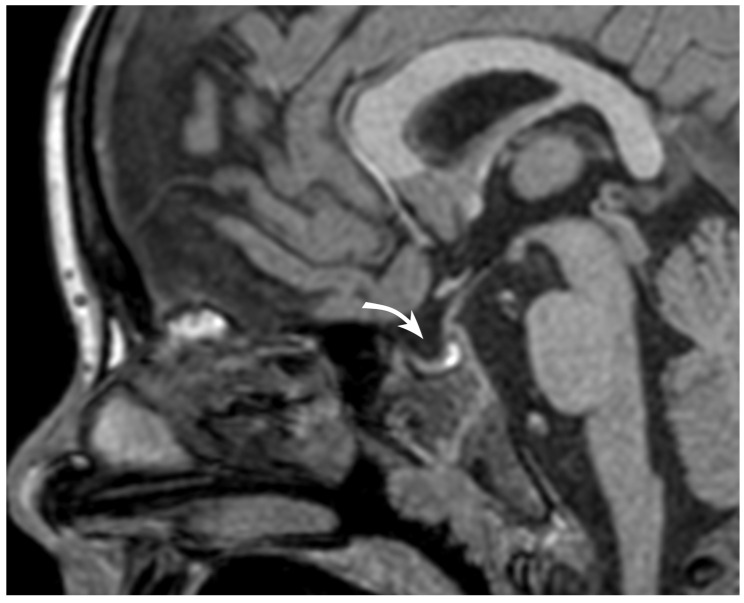
The sagittal T1 sequence shows the sella turcica filled by CSF with a distinct concavity of the hypophysis.

**Figure 5:**
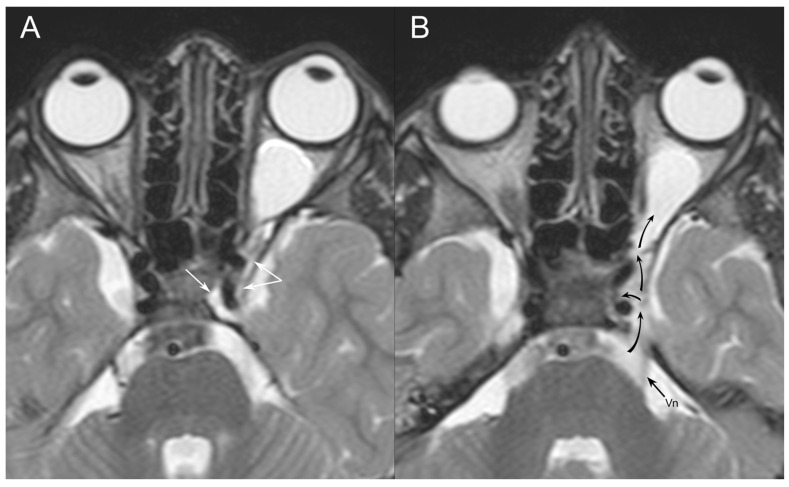
In the two T2 axial planes, the CSF signal is detected within the cavernous sinus, both medially and laterally to the internal carotid artery (white arrows on (**A**)). (**B**): The cisternal segment of the trigeminal nerve is eccentric with respect to the Meckel cave. A separation of the Meckel cave and cavernous sinus is not detectable, and the path of the CSF signal runs toward the superior orbital fissure ending in the intraorbital meningocele (black arrows).

In the following days, clinical deterioration was observed with increasing lethargy, vomiting, worsening of bilateral papilledema, left eyelid ptosis, reduction in visual acuity (8/10 on the left side), minimal limitation in upper rotation of the left eye, and marked limitation in lateral rotation muscle of the right eye. A new contrast-enhanced angio-MR was performed (Figure 4 and Figure 5) that demonstrated a mild progression of the thrombosis alongside radiological signs of ICH including distended ON sheaths, edematous signal changes in the ONs, and empty sella. 

**Figure 6:**
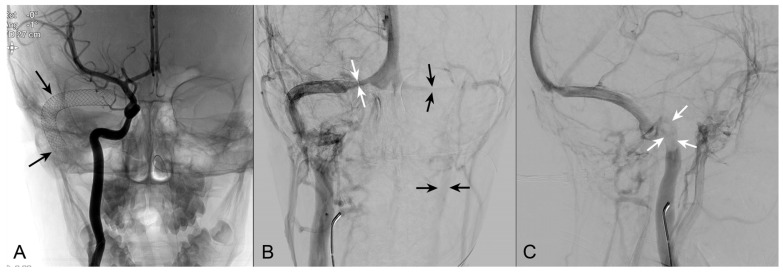
Arteriography with venous phase. (**A**): Selective injection in the right ICA, a stent has been placed inside the right transverse sinus. (**B**): Patency of the stent is demonstrated. Residual stenosis of the right transverse sinus at the origin (white arrows). Hypoplastic left transverse sinus with faint contrast agent filling of the left jugular vein (black arrows). (**C**): The oblique view demonstrates a residual thrombus at the origin of the right jugular vein.

After neuroradiological consultation, the patient underwent staged endovascular treatment (with a 1-week interval). In the first step, the right transverse and sigmoid sinuses were recanalized and a self-expandable stent was implanted to maintain a sufficient flow. A second procedure was necessary after 7 days of dual antiplatelet therapy (DAPT) and heparin due to persistent symptoms related to ICH (dizziness, cephalalgia, and left meningocele). During this procedure, intravenous manometry of the superior sagittal and transverse sinuses demonstrated a remarkable intra-extracranial pressure gradient that required the placement of a second stent into the proximal transverse sinus and angioplasty of the organized clot in the jugular bulb (Figure 6). This led to a progressive improvement in clinical conditions.

**Figure 7:**
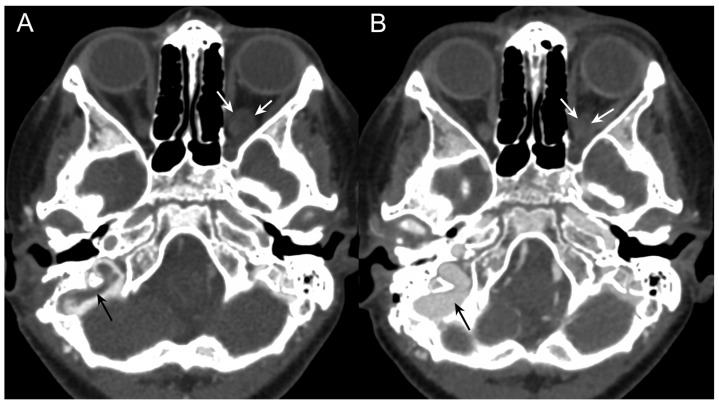
(**A**): CT obtained a few days after placement of the right transverse sinus stent shows residual thrombus (black arrow) and a reduction in the size of the intraorbital meningocele (white arrows). (**B**): At follow-up CT acquired after one month, complete patency of sigmoid sinus is achieved (black arrow). A further reduction in the size of the meningocele is shown (white arrows).

Ten days after the procedure, a contrast-enhanced CT scan showed a reduction of the orbital MC (from 26 × 20 mm to 18 × 9 mm), along with perfusion of the transverse and sigmoid sinuses and the proximal jugular vein with residual thrombus (Figure 7a). At the time of discharge, a mild deficit in the elevation of the left eye, in the right lateral rectus muscle function, and left eyelid ptosis persisted. Low molecular weight heparin (LMWH) therapy for 3 months and DAPT for 6 months were indicated. Visual function and orthoptic findings normalized within 1 week after discharge. The contrast-enhanced CT performed at 1 month after discharge (Figure 7b) showed no residual signs of venous thrombosis and a further reduction of the orbital MC (12 × 5 mm). 

**Figure 8:**
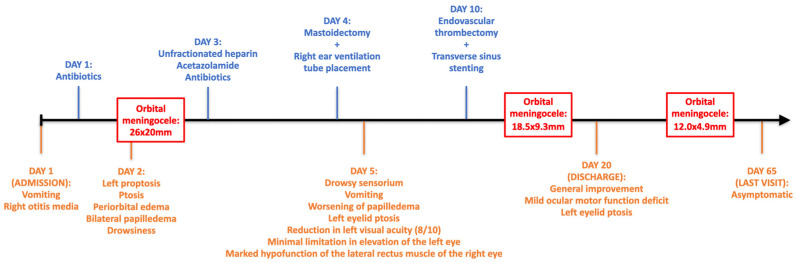
The clinical course of the patient.

The latest clinical evaluation (after the 3 month follow-up) revealed a return to general baseline conditions, with the patient being asymptomatic. The subsequent contrast-enhanced MRI was scheduled within six months. The clinical course is summarized in Figure 8.

### 3.2. Systematic Review

**Figure 9:**
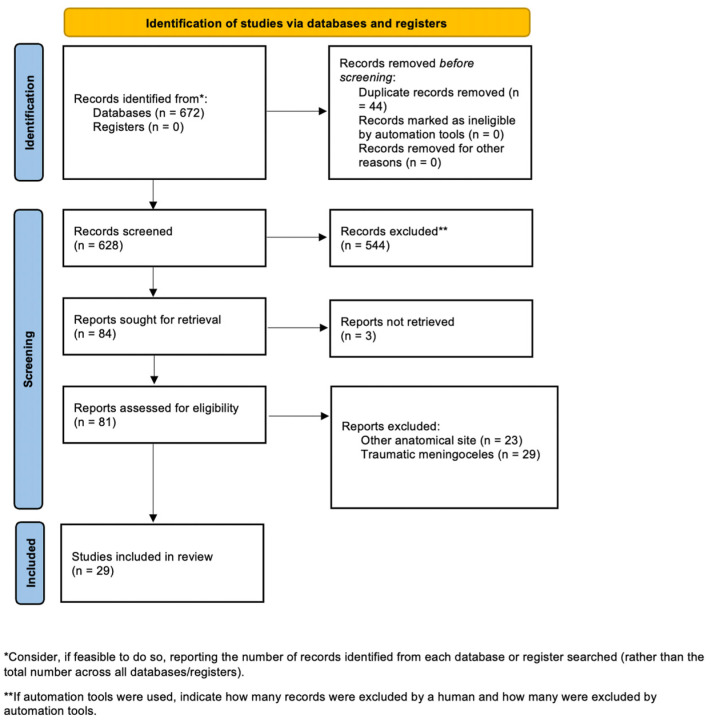
PRISMA flowchart illustrating the article selection process.

The literature search retrieved 628 studies. Based on exclusion criteria, 597 of the screened studies were excluded, according to PRISMA guidelines (Figure 9).

The details of the studies that met inclusion and exclusion criteria are shown in Table 1. 

**Figure 10:**
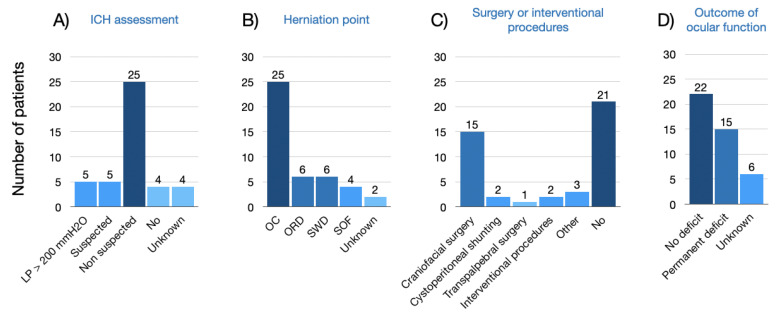
Bar plots of general characteristics of the systematic review: (**A**) ICH assessment; (**B**) herniation point; (**C**) surgery or interventional procedures performed; (**D**) outcome of ocular function.

The systematic review included a total of 29 studies published from 1981 to 2023, reporting on 43 patients affected by spontaneous orbital cephalocele. The clinical details are summarized in Table 1 and Table 2 and in Figure 10. 

Including the case report presented herein, 34 (79.1%) patients were affected by MC and 9 (20.9%) by MEC. The ages ranged from 15 days to 82 years, while 18 (41.9%) were pediatric patients and 25 (58.1%) were adults. In 25 cases (58.1%), the MC herniated through the optic canal (OC) making it the most frequently observed point of herniation, followed by orbital roof defects (ORD), greater sphenoidal wing defects (GSWD), and SOF. Moreover, the OC herniation point was the only one described as bilateral (15 cases, 34.9%). The most commonly reported symptom was loss of vision (22 cases, 51.2%), followed by proptosis (11 cases, 25.6%), orbital content displacement (8 patients, 18.6%), periorbital swelling (8 cases, 18.6%), headache (6 patients, 13.9%), orbital pulsation (4 cases, 9.3%), orbital pain (3 patients, 7.0%), and CSF leak (1 case, 2.3%). Only two patients (4.7%) did not complain of any symptoms, as orbital MC was an incidental finding. 

ICH was investigated by lumbar puncture (LP) in nine patients (20.9%): in five (11.6%) the pressure was >200 mmH2O, while in four (9.3%) outflow pressure was in the normal range. In five cases (11.6%), ICH was suspected based on clinical evaluation (headache, vomiting, abducent nerve palsy, papilledema, etc.) and radiological signs (e.g., empty sella or ON sheath dilatation), but LP was not performed. Of note, five patients (11.6%) had a documented history of neurofibromatosis type 1 (NF1), while in two patients (4.7%) NF1 was only suspected for the presence of café-au-lait spots and/or family history.

Surgical treatment was performed in 21 (48.8%) patients, of whom 15 (34.9%) received craniofacial surgery (such as bifrontal or frontotemporal craniotomy with orbital roof reconstruction). Three patients (7.0%) received a procedure aimed to reduce the ICH: two (4.7%) patients underwent cystoperitoneal shunting (one of these procedures was concomitant to craniofacial surgery) and one (2.3%) received thrombectomy with transverse sinus stenting (our case report). Both interventions led to improvement in ocular function (visual acuity and/or ocular motor function). Seven (16.3%) patients received treatment with acetazolamide only, which resulted in improvement in ocular function in 5 cases (11.6%). In the other two cases (4.7%), visual impairment persisted but did not worsen. In over half of the patients (22, 51.2%), no residual visual deficit was observed, while in 15 cases (34.9%) a permanent visual deficit (diplopia or loss of vision) was reported during follow-up.

## 4. Discussion

Spontaneous orbital cephalocele is a rare finding, with only 43 cases reported in the literature. The complexity of this condition highlights the necessity for a comprehensive multidisciplinary approach in both diagnosis and treatment. The systematic review presented herein revealed considerable heterogeneity in demographic characteristics, diagnostic features, and management strategies reported in the literature. Of note, the most frequent presentation described was spontaneous MC herniated through the OC, which was bilateral in more than half of patients. This finding is likely attributable to the fact that the ON sheath is an extension of all meningeal layers, thus facilitating herniation of intracranial neural tissue [41]. This interpretation aligns with the fact that MCs herniated through the OC are the only ones documented in the literature with LP opening pressure exceeding 200 mmH2O, supporting the rationale to measure ON sheath diameter as a diagnostic criterion for ICH [42,43].

Orbital cephalocele is usually a symptomatic condition, with only 2 (4.7%) incidental diagnoses. Typically, manifestations include loss of vision, proptosis, displacement of orbital content, periorbital swelling, headache, orbital pulsation, or orbital pain (Table 3). According to the systematic review presented in this article, loss of vision emerged to be more characteristic when the herniation occurs from the OC, whereas proptosis, orbital pulsation, and orbital content displacement were more typical of herniations from ORD or GSWD. In MCs/MECs herniated from the SOF, the most reported symptom was periorbital swelling, followed by proptosis and orbital content displacement (as in our case). This trend is consistent with the site of herniation: the increased occurrence of visual symptoms in OC MCs can be attributed to ON compression and ischemia, whereas periorbital swelling in MCs herniated through the SOF may be a result of compression on the superior ophthalmic vein. Interestingly, NF1 was more frequently diagnosed or suspected in patients with MC herniation from OC or GSWD. Skull base or spine MCs are described as characteristic (even if not pathognomonic) elements of NF1 [44], with frequent association with greater sphenoidal wing dysplasia (occurring in 4–11% of NF1 patients) [45]. The latter can result in the widening of orbital fissures, possibly playing a role in the herniation of MC/MEC [46,47].

The different experiences reported in the literature highlight the need for personalized approaches, tailored by a multidisciplinary group (involving head and neck surgeons, neurosurgeons, ophthalmologists, radiologists, and neuroradiologists) considering individual patient factors and disease-specific features. Surgical treatment is frequently reported in the literature, but conservative options such as watch-and-scan schedule [9,10] or medical therapy with acetazolamide [11,12] have been reported. Among surgical strategies, craniotomy or transpalpebral transorbital approaches have been described [13,14]. CSF leak is the most frequently reported postoperative complication [5]. 

In the case report presented herein, the role of ICH was deemed to be paramount in the pathogenesis of the MC herniation through the SOF. This was corroborated by the finding of bilateral transverse sinus disease, with the left side affected by congenital hypoplasia, and the other side by an acute thrombotic event. The pivotal role of transverse sinus stenosis in increasing intracranial pressure, particularly when bilateral, has been previously proposed in the literature [48,49,50,51]. Our case report aligns with this evidence. Currently, LP is the gold standard for diagnosis of ICH, and treatment may involve lumbar drainage (LD) [52]. However, in our case, ICH was suspected due to bilateral transverse sinus stenosis (one with thrombosis, the other with hypoplasia), accompanying symptoms, and radiological signs. Currently, MCs are not considered a strong diagnostic criterion for ICH, even in the case of transverse sinus thrombosis [53,54,55,56,57]. Nonetheless, in our case, anticoagulant treatment for transverse sinus thrombosis was preferred in place of LP or ventricular drainage, following a pathophysiological approach guided by the suspicion of ICH being determined by insufficient venous return [52]. The hypothesized mechanism of orbital MC formation appeared to be validated by its reduction after transverse sinus thrombectomy, stenting, and acetazolamide administration. This treatment not only targeted the pathogenic mechanisms contributing to ICH but also shed light on the potential reversibility of the associated MC by treating its underlying cause. The efficacy of this combined therapeutic approach corroborates the importance of understanding and addressing the underlying pathophysiological factors in the management of spontaneous orbital MC. Despite the theories supporting the role of ICH in the development of orbital cephaloceles [11,21,51,58], many previous experiences have poorly explored this aspect. Only nine studies in the literature reported diagnosis of ICH using radiologic or instrumental investigations, reflecting a gap in the understanding of orbital MC. This could have led to missing a diagnosis of ICH, opting for surgical treatment directly addressing the MC and its herniation point (such as a craniofacial approach with MC resection and orbital roof plasty [32] or frontotemporal craniotomy and repair of bone defect with partial thickness calvaria autologous bone graft [14]) with no investigation of the pathophysiological mechanism. The finding of ICH in orbital MC can support a more pathophysiology-oriented treatment approach to the underlying cause of the MC. However, the clinical presentation of intraorbital MC/MEC is heterogeneous, and at times the underlying causes remain concealed.

The current study has some limitations. The small number of patients documented in the literature precludes a meta-analysis, and the limited available data constrains a more in-depth investigation into the pathophysiological mechanisms of MC/MEC formation. Notwithstanding these limitations, we believe that the case report and insights from the systematic review should promote a more comprehensive investigation of the pathophysiology-oriented treatment modalities for spontaneous orbital cephaloceles.

## 5. Conclusions

This study emphasizes the heterogeneous presentation of spontaneous orbital cephaloceles and the complexity of their management. The results stress the importance of a multidisciplinary and individualized approach to diagnosis and management. The insights from both the systematic review and the case report support the importance of considering ICH and associated anatomical factors in the management of this rare pathological condition. Moreover, the the importance and possible advantages of treating the underlying cause of spontaneous orbital cephaloceles are highlighted.

## Figures and Tables

**Table 1:** Studies included in the systematic review.

Publication (First Author, Year)	Number of Cases	Age	Gender	Side	Diagnosis	Ich	Erniation Point Identification	Surgery or IP	Medical Treatment for Ich	Associated Medical Conditions
Weizman, 1981 [19]	1	15 days	F	U	MC	Non susp	ORD	Yes	Yes	
Downey, Jr, 1983 [10]	1	1 year	M	U	MEC	Non susp	SOF	No	No	Contralateral retinoblastoma
Bornemann, 1988 [20]	1	Pediatric NOS	M	U	MEC	Non susp	SOF	Yes		
Garrity, 1990 [21]	13	3–61 years (3 pediatrics NOS)	7M 6F	11 B 2 U	13 MC	2 Yes 1 Susp 10 Non susp	13 OC	3 Yes 10 No		3 NF1
Sugawara, 1995 [22]	1	5 years	M	U	MEC	Non susp	GSWD	Yes		Suspected NF1
Lunardi, 1997 [23]	1	15 years	M	U	MC	No	OC	Yes		
Shanmuganathan, 2002 [24]	1	59 years	M	B	MC	Yes	OC	No		
Mesa-Gutiérrez, 2008 [25]	1	53 years	M	U	MC	Yes	OC	No	Acetazolami (250 mg ×2)	
Knopp, 2009 [26]	1	4 days	M	U	MEC	Non susp	GSWD	Yes		
Spooler, 2009 [27]	1	5 years	M	U	MC	Susp	OC	Yes		Multiple malformations
Ahmed, 2012 [28]	1	Prenatal period	F	U	MEC		SOF	Yes	No	
Halimi, 2013 [29]	1	39 years	F	U	MC	Susp	OC	No	Acetazolami (125 mg ×2–3)	
Sharma, 2014 [30]	1	22 years	M	U	MEC	Non susp	GSWD	Yes		
Germano, 2015 [31]	1	51 years	F	U	MC	Non susp	ORD	Yes		
Trivedi, 2015 [14]	1	10 years	F	U	MC	No	GSWD	Yes		Suspected NF1
Van Rumund, 2017 [3]	1	79 years	M	U	MC	Non susp	ORD	No		
Hoang, 2017 [32]	1	3 years	F	U	MEC	Non susp	ORD	Yes	No	
Mahatma, 2017 [33]	1	10 years	M	B	MC	Susp	OC	Yes		
Sioufi, 2017 [9]	1	53 years	M	B	MC	Non susp	OC	No	No	Left choroidal melanoma
Algarni, 2018 [34]	2	82 years 53 years	1 M 1 F	2 U	2 MC	1 Yes 1 No	2 OC	2 No		
Jain, 2019 [12]	1	13 years	F	U	MC	Non susp	OC	No	Acetazolami (250 mg)	
Shai kh, 2019 [35]	1	20 years	F	U	MC	Non susp	OC	No		NF1
Zurita, 2020 [36]	1	41 years	F	U	MEC	Non susp	No	Yes		
Mbaye, 2020 [37]	1	7 years	F	U	MC	Non susp	No	Yes	Acetazolami (250 mg)	
Kulkarni, 2021 [38]	1	20 years	M	U	MC		ORD	Yes		
Peto, 2021 [13]	1	50 years	M	U	MEC		ORD	Yes		
Lai, 2021 [39]	1	41 years	F	U	MC		GSWD	Yes		NF1
Morello, 2022 [11]	1	46 years	F	B	MC	No	OC	No	Acetazolami (250 mg ×2)	
Rajabi, 2023 [40]	1	4 years	F	U	MC	Non susp	GSWD	Yes		
Present case report	1	6 years	M	U	MC	Susp	SOF	Yes	Acetazolami(250 mg)	
Overall	43	15 days–82 years	23 M 20 F	28 U 15 B	34 MC 9 MEC	5 Yes 5 Susp 25 Non susp 4 No	25 OC 6 ORD 6 GSWD 4 SOF	22 Yes 21 No	7 Yes 4 No	7 NF or suspected 3 other 30 no

IP: interventional procedures; M: male; F: female; NOS: not otherwise specified; U: unilateral; Ich: intracranial hypertension; Susp: suspected; Non susp: non suspected; OC: optic canal; ORD: orbital roof defect; SOF: superior orbital fissure; GSWD: greater sphenoidal wing defect; MC: meningocele; MEC: meningoencephalocele.

**Table 2:** Characteristics of patients included in the systematic review.

	N (% or CI)
Sex	
Male	23 (53.5%)
Female	20 (46.5%)
Median age in years (range)	27.7 (20.3–35.1)
ICH assessment	
LP > 200 mmH2O	5 (11.6%)
Suspected	5 (11.6%)
Non suspected	25 (58.1%)
No	4 (9.3%)
Unknown	4 (9.3%)
Surgery or interventional procedure	
Surgery	21 (48.8%)
Craniofacial surgery	15 (71.4%)
Cystoperitoneal shunting	2 (9.5%)
Transpalpebral surgery	1 (4.8%)
Other	3 (14.3%)
Interventional procedure	1 (2.4%)
No	21 (48.8%)
Herniation point	
Optic canal	25 (58.1%)
Unilateral	15 (60.0%)
Bilateral	10 (40.0%)
Orbital roof defect	6 (13.9%)
Greater sphenoidal wing defect	6 (13.9%)
Superior orbital fissure	4 (9.3%)
Unknown	2 (4.8%)
Medical treatment for ICH (Acetazolamide)	
Yes	7 (16.3%)
No	5 (11.5%)
Unknown	31 (72.2%)
Outcome for ocular function	
No deficit (diplopia or visual acuity)	22 (51.2%)
Permanent deficit (diplopia or visual acuity)	15 (34.9%)
Unknown	6 (13.9%)
Associated medical conditions	
NF1 or suspected NF1	7 (16.3%)
Others	3 (7.0%)
None	33 (76.7%)
Median follow-up (months)	9.9 (reported only in 14 cases)

ICH: intracranial hypertension; LP: lumbar puncture; CI: confidence interval; NF1: neurofibromatosis 1.

**Table 3:** Analysis of symptoms, intracranial hypertension, and association with neurofibromatosis 1 with the herniation point of the cephalocele.

Herniation Point	Symptoms	ICH	NF1
Optic canal (25)	18 (72.0%) Loss of vision 8 (32.0%) Headache 2 (8.0%) Orbital content displacement 2 (8.0%) Proptosis 2 (8.0%) Orbital pain 1 (4.0%) Asymptomatic	5 (20.0%) PL > 200 mmH2O 4 (16.0%) suspected 14 (56.0%) Not suspected 2 (8.0%) No	4 (16.0%) Yes 21 (84.0%) No
Orbital roof defect (6)	3 (50%) Periorbital swelling 3 (50.0%) Proptosis 3 (50%) Orbital pulsation 1 (16.7%) Loss of vision 1 (16.7%) Orbital content displacement 1 (16.7%) Headache 1 (16.7%) Liquorrea	4 (66.7%) Not suspected 2 (33.3%) Unknown	6 (100%) No
Greater sphenoidal wing defect (6)	5 (83.3%) Proptosis 3 (50%) Orbital pulsation 3 (50%) Orbital content displacement 2 (33.3%) loss of vision 1 (16.7%) Orbital pain	4 (66.7%) Not suspected 1 (16.7%) No 1 (16.7%) Unknown	1 (16.7%) Yes 2 (33.3%) Suspected
Superior orbital fissure (4)	3 (75.0%) Periorbital swelling 2 (50.0%) Proptosis 1 (25.0%) Orbital content displacement 1 (25.0%) Asymptomatic	2 (50.0%) Not suspected 1 (25.0%) Suspected 1 (25.0%) Unknown	4 (100%) No

ICH: intracranial hypertension; NF1: neurofibromatosis type 1.

## Data Availability

The raw data supporting the conclusions of this article will be made available by the authors upon request.

## Supplementary Materials

The following supporting information can be downloaded at: https://www.mdpi.com/article/10.3390/jpm14050465/s1, Table S1: Assessment of ethical compliance and completeness in the presentation of the 29 selected articles in accordance with the Joanna Briggs Institute (JBI) checklist.
