# Supplementary material for: A Pathophysiological Approach to Spontaneous Orbital Meningoceles: Case Report and Systematic Review
Source: J Pers Med. 2024 Apr 28;14(5):465. doi: 10.3390/jpm14050465 (PMC11122061; doi:10.3390/jpm14050465)
Supplement: Supplementary file 1 [file jpm-14-00465-s001.zip › jpm-2938835-supplementary.pdf]

Supplementary Table S1. Assessment of ethical compliance and completeness in the presentation of the 29 selected articles in accordance with the Joanna Briggs Institute (JBI) checklist.

	**Yes**	**No**	**Unclear**	**Not applicable**
Were patient's demographic characteristics clearly described?	28	1		
Was the patient's history clearly described and presented as a timeline?	3	23	3	
Was the current clinical condition of the patient on presentation clearly described?	25	3	1	
Were diagnostic tests or assessment methods and the results clearly described?	25	2	2	
Was the intervention(s) or treatment procedure(s) clearly described?	17	6	1	5
Was the post-intervention clinical condition clearly described?	16	7	1	5
Were adverse events (harms) or unanticipated events identified and described?	9	13	1	6
Does the case report provide takeaway lessons?	14	10	5	
**Overall appraisal**	**Include**	**Exclude**	**Seek further info**	
	29	0	0	
